# A Donkey Blood-Derived Bioactive Peptide (YPWTQ) Alleviates Insulin Resistance in HepG2 Cells Through Multi-Target Regulation of Glucose and Lipid Metabolism and Oxidative Stress

**DOI:** 10.3390/nu18152445

**Published:** 2026-07-27

**Authors:** Qian Zhang, Xiaotong Wu

**Affiliations:** School of Life Sciences, Inner Mongolia University, Hohhot 010070, China

**Keywords:** donkey blood-derived hypoglycemic peptide, nutritional supplements, insulin resistance, type 2 diabetes, multi-omics

## Abstract

Background: Type 2 diabetes (T2DM) is a chronic metabolic disease closely associated with insulin resistance (IR) and disturbances in glucose and lipid metabolism. Bioactive peptides derived from food are attracting increasing research attention as candidates for nutritional supplements or functional food ingredients that improve metabolic health. This study evaluated the functional food-related properties of YPWTQ (CP4), a novel peptide derived from donkey blood, and its ability to alleviate insulin resistance in HepG2 cells. Methods: CP4 was characterized based on its hemolytic activity, stability under simulated gastrointestinal digestion conditions, inhibitory activity against α-glucosidase and Pancreatic lipase, and free radical scavenging capacity (DPPH·, ABTS^+^·, and O_2_^−^·). Its effects on glucolipid metabolism and oxidative stress were examined in a glucosamine-induced insulin-resistant HepG2 cell model. Candidate signaling pathways associated with CP4 treatment were explored through transcriptomic and metabolomic analyses, combined with RT-qPCR technology. Results: CP4 exhibited low hemolytic activity and remained stable after 4 h of simulated gastrointestinal digestion. It inhibited α-glucosidase and Pancreatic lipase and exhibited antioxidant activity. In insulin-resistant HepG2 cells, CP4 increased glucose consumption, glycogen content, and cell survival, while reducing triglyceride accumulation, malondialdehyde levels, and reactive oxygen species (ROS) production. Mult omics analysis indicates that these phenotypic effects may be associated with coordinated changes in the PI3K-Akt, AGE-RAGE, Rap1, and Ras signaling pathways, as well as related genes and metabolites. Conclusions: These findings suggest that CP4, as a food-derived bioactive peptide candidate, warrants further investigation into its potential applications in the field of metabolic health.

## 1. Introduction

Type 2 diabetes mellitus (T2DM) is a chronic metabolic disease characterized by persistent disruption of glucose and lipid homeostasis, with insulin resistance (IR) now recognized as a core driving factor in both disease progression and diabetic complications [1]. Although current pharmacological therapies can improve glycemic control, they are frequently associated with adverse effects such as hypoglycemia, weight gain, and hepatotoxicity, and their efficacy often diminishes over time [2]. These limitations have spurred increasing interest in safer, food-derived bioactive compounds as potential nutraceutical or functional food ingredients for T2DM management [3].

In research on blood glucose control, bioactive peptides from various sources have been shown to possess significant activity. Based on their sources, anti-diabetic bioactive peptides are widely found in natural substances such as animals, plants, and microorganisms; based on their mechanisms of action, natural blood glucose-lowering peptides can be primarily classified into DPP-IV-inhibiting peptides, α-glucosidase-inhibiting peptides, and α-amylase-inhibiting peptides, among others [4].

Among the various natural sources, animal blood has gradually attracted the attention of researchers due to its high content of high-quality protein. Animal blood, a byproduct of livestock and poultry processing, is rich in high-quality protein and has become an ideal raw material for the preparation of bioactive peptides [5]. In recent years, bioactive peptides derived from animal blood sources such as camel blood [6], bovine blood [7], and chicken blood [8] have been extensively studied. These peptides exhibit various physiological activities, including antioxidant, lipid-lowering, and blood-glucose-lowering effects [9]. With the continuous advancement of high-value utilization of food resources and the concept of green processing, the transformation of animal blood from traditional waste to a high-value-added functional ingredient has become an important research direction in the field of livestock product processing. Among these, donkey blood—a byproduct of the donkey breeding industry—has long been underutilized; most of it is discarded or undergoes only simple processing, resulting in a waste of resources [10]. In recent years, with the continuous advancement of research in food nutrition and bioactive substances, the nutritional and medicinal value of donkey blood has gradually come to light. Donkey blood is rich in protein and trace elements; data show that its protein content in dry matter exceeds 90%, with hemoglobin accounting for 60–65%, laying a solid material foundation for its use as a source of bioactive peptides [11]. Currently, research on bioactive peptides in donkey blood is relatively scarce; however, existing studies have confirmed that enzymatic hydrolysates of donkey blood possess various physiological activities, such as antioxidant, antihypertensive, and anti-fatigue effects [12]. Nevertheless, research on the role of bioactive peptides derived from donkey blood in regulating glucose metabolism remains limited, with a particular lack of systematic studies focused on improving insulin resistance (IR). Liu Dan purified Component III from the <3 kDa ultrafiltration fraction of donkey blood hemoglobin hydrolysate and identified 16 hypoglycemic peptides via LC-MS/MS [13]. However, the hypoglycemic activity of these 16 peptides varies in strength, and their hypoglycemic effects at the cellular level, as well as their mechanisms of action in alleviating insulin resistance, remain unclear. The YPWTQ (CP4) used in this study is derived from one of these 16 peptides and, based on molecular docking predictions, exhibits good hypoglycemic potential. Systematic screening and evaluation of hypoglycemic peptides derived from donkey blood will not only enrich research on bioactive peptides from animal blood sources but, more importantly, provide new avenues for the high-value utilization of donkey blood by-products and establish a foundation of candidate materials for the screening, stockpiling, and development of natural hypoglycemic factors.

The liver is a central organ for glucose production, glycogen synthesis, lipid metabolism, and insulin action. Accordingly, the glucosamine-induced insulin-resistant HepG2 cell model is widely employed to evaluate candidate compounds with the potential to ameliorate hepatic IR [14]. Nevertheless, HepG2 cells are derived from hepatocellular carcinoma and may differ from primary hepatocytes in insulin signaling, gluconeogenic response, and broader metabolic phenotype [15,16]. Thus, this model is useful for exploratory evaluation, but it cannot fully reproduce the complexity of human T2DM pathophysiology. Furthermore, integrated multi-omics strategies—combining transcriptomics and metabolomics—can prioritize convergent signaling and metabolic pathways, offering mechanistic insights beyond the simple enumeration of differentially expressed genes and metabolites [17].

In the present study, we combined comprehensive in vitro characterization with the glucosamine-induced IR-HepG2 model and an integrated multi-omics analysis to systematically evaluate CP4. Specifically, we assessed hemolytic activity, simulated gastrointestinal stability, inhibitory activities against α-glucosidase and pancreatic lipase, and antioxidant properties as indicators of its suitability for functional food applications. We then determined whether CP4 could ameliorate the insulin-resistant phenotype in HepG2 cells. Finally, by integrating transcriptomics, metabolomics, and RT-qPCR validation, we proposed hypotheses regarding candidate signaling pathways. Given the exploratory nature of this model, we identified the PI3K-Akt, AGE-RAGE, Rap1, and Ras pathways as relevant signaling pathways, these pathways require further validation at the protein level and in vivo.

## 2. Materials and Methods

### 2.1. Materials

The 6–8-week-old C57BL/6 was purchased from Sperfo Biotechnology Co., Ltd. (Beijing, China). Pepsin, trypsin, α-glucosidase, and pancreatic lipase were purchased from Yuanye Biotechnology Co., Ltd. (Shanghai, China). CP4 and FITC-labeled CP4 were custom-synthesized by Nanjing Taopu Biotechnology Co., Ltd. (Nanjing, China). The reactive oxygen species (ROS), Actin-Tracker Red, 4′,6-diaminobenzidine (DAPI) staining kits, and glucosamine were purchased from Bioyate Biotechnology (Shanghai, China). The malondialdehyde (MDA) quantitative kit was obtained from Greys Biotechnology (Shanghai, China). The BCA protein quantitative kit was provided by Saifu Biotechnology (Wuhan, China), and the SYBR Green qPCR super mixture was supplied by Yali Biotechnology Co., Ltd. (Suzhou, China). The detection kits for glucose, glycogen, and triglyceride (TG) determination were from Nanjing Jiancheng Biotechnology Institute (Nanjing, China). The human HepG2 cell line was stored in the laboratory.

### 2.2. Evaluation of Hemolysis, Stability and In Vitro Biological Activity of CP4

#### 2.2.1. Hemolysis of CP4

This experimental protocol has been approved by the Bioethics Committee of Inner Mongolia University (Approval No. 129, 2024; Approval Date: 25 November 2024). C57BL/6 mice underwent a one-week acclimatization period at the Laboratory Animal Breeding Center of the School of Life Sciences at Inner Mongolia University. The hemolysis test was modified slightly based on the method of Li et al. [18]. CP4 was dissolved in sterile water to obtain solutions with concentrations of 1 mg/mL and 2 mg/mL. Mouse venous blood was collected, washed three times with pH 7.4 PBS, and then centrifuged at 1500 r/min for 15 min at 4 °C. The precipitated red blood cells were diluted to a 4% cell concentration with PBS buffer and mixed with the CP4 solution. The mixture was incubated at 37 °C for 1 h and then centrifuged at 4 °C, 1000 r/min. The supernatant was taken to measure its absorbance at 595 nm. The negative control was PBS buffer, and the positive control was 0.1% TritonX-100. The hemolysis rate of CP4 was calculated as follows:

$$Hemolysis\;rate(\%)=\frac{A-A_{0}}{A_{1}-A_{0}}\times 100\%$$

where *A* represents the absorbance value (OD595) of CP4; *A*_0_ represents the absorbance value (OD595) of PBS buffer; and *A*_1_ represents the absorbance value (OD595) of 0.1% TritonX-100.

#### 2.2.2. Gastrointestinal Stability of CP4

The simulation of gastrointestinal stability was conducted by slightly modifying the method of Wu Tong [19]. CP4 was dissolved in sterile ultrapure water, then gastric protease (enzyme: substrate = 1:10) was added. An amount of 1.5 mL was used as a blank. The pH was adjusted to 2, and then the mixture was incubated at 37 °C for 2 h. Every 1 h, 1.5 mL was taken and the pH was adjusted to 7. The solution was boiled in water bath for 5 min to inactivate the enzyme, then filtered through a 0.22 μm filter membrane and analyzed on the machine. After 2 h, the samples were taken out and the pH was adjusted to 7. Pancreatic protease (enzyme: substrate = 1:10) was added and then incubated at 37 °C for 2 h. Every 1 h, 1.5 mL was taken and the pH was adjusted to 7. The solution was boiled in a water bath for 5 min to inactivate the enzyme, then filtered through a 0.22 μm filter membrane and analyzed on the machine. The specific detection parameters are shown in Table 1. By comprehensively analyzing the retention times and peak areas of the liquid chromatogram, the degradation of CP4 under simulated gastrointestinal conditions was determined.

#### 2.2.3. In Vitro Functional Activity of CP4

(1) α-Glucosidase inhibition rate

We weighed 0.03 g of pNPG (4-nitrophenyl-α-D-xylopyranoside) and dissolved it in phosphate sodium salt buffer by ultrasound and adjusted the volume to 50 mL to prepare a 2 mM pNPG solution. Also, we weighed 4.24 g of anhydrous Na_2_CO_3_ and dissolved it in distilled water to adjust the volume to 200 mL. After ultrasonic treatment, a 0.2 M Na_2_CO_3_ solution was obtained. We added 40 μL of CP4 sample solution and 40 μL of 1 U/mL α-glucosidase solution to each reaction well in sequence, mixed well, and pre-incubated the mixtures at 37 °C for 5 min. After pre-incubation, we immediately added 40 μL of 2 mM pNPG substrate solution to each well, mixed well, and continued incubation at 37 °C for 30 min. After the above enzymatic reaction, we immediately added 80 μL of 0.2 M Na_2_CO_3_ to terminate the reaction, mixed well, and detected the OD value of the reaction system at 405 nm wavelength using an enzyme detector. The sample group is designated as *A*_1_; the blank group, in which the sample is replaced with PBS buffer, is designated as *A*_2_; and the control group, in which α-glucosidase is replaced with PBS buffer, is designated as *A*_0_. The positive control was prepared by dissolving acarbose solution in 50% DMSO to replace the CP4 solution. Each group had 3 replicates. The α-glucosidase inhibition rate of CP4 was calculated as follows:

$$\alpha -glucosidase\;inhibition\;rate(\%)=(1-\frac{A_{1}-A_{0}}{A_{2}-A_{0}})\times 100\%$$

where *A*_1_ is the sample group, *A*_2_ is the sample blank group, and *A*_0_ is the sample control group.

(2) Pancreatic lipase inhibition rate

The determination of pancreatic lipase inhibition rate was based on the method of Wan Lin [20]. Prepare separate solutions of 5 mg/mL pancreatic lipase, buffer (pH 8.2, 0.1 mol/L Tris buffer), and reaction substrate (1% Triton X-100, 5 mmol/L sodium acetate aqueous solution, 1 mg/mL lauric acid-4-nitrophenyl ester). To each sample well, add 200 μL of sample solution, 500 μL of substrate solution, 400 μL of buffer, and 300 μL of pancreatic lipase solution in that order. The samples were incubated in a 37 °C incubator for 2 h. The reaction mixture was then centrifuged, and the supernatant was collected to measure the absorbance at a wavelength of 420 nm. For the positive control group, an orlistat solution was used in place of the sample solution. Three replicates were performed for each group. The pancreatic lipase inhibition rate was calculated using the following formula:

$$Pancreatic\;lipase\;inhibition\;rate(\%)=(1-\frac{A-B}{C})\times 100\%$$

in the equation, *A* is the absorbance of the sample group; *B* is the absorbance of the group without lipase; and *C* is the absorbance of the group without a sample.

(3) DPPH· scavenging rate

DPPH was dissolved in ethanol to prepare a 0.2 mM DPPH solution. The following steps were used: mix 50 μL of different concentration of peptide solution with 50 μL of DPPH solution and record as *A_i_*; mix the peptide solution with anhydrous ethanol and record as *A_j_*; and mix the DPPH solution with anhydrous ethanol and record as *A*_0_. Each reaction solution was shaken and mixed well, sealed, and reacted at room temperature in the dark for 30 min, then measured at 517 nm wavelength. Each group had 3 replicates, and vitamin C (VC) was used as the positive control. The DPPH· scavenging rate of CP4 was calculated as follows:

$$DPPH\cdot \;scavenging\;rate(\%)=1-\frac{A_{i}-A_{j}}{A_{0}}\times 100\%$$

(4) ABTS^+^· scavenging rate

Mix 7 mM ABTS solution with 2.45 mM potassium persulfate solution in equal volumes and let it react at room temperature in the dark for 16 h to prepare the ABTS working solution. Before use, dilute it to an absorbance of 0.70 ± 0.02 at a wavelength of 734 nm. During the experiment, mix 50 μL of peptide solutions of different concentrations with 50 μL of the ABTS working solution and label it as *A_i_*; mix the peptide solution with PBS and label it as *A_j_*; and mix PBS with the ABTS working solution and label it as *A*_0_. After thorough mixing of all components, let them react in the dark for 30 min and measure the OD value at 734 nm. Each group has 3 replicates, and VC is used as the positive control. Calculate the CP ABTS^+^· scavenging rate as follows:

$${ABTS}^{+}\cdot \;scavenging\;rate(\%)=1-\frac{A_{i}-A_{j}}{A_{0}}\times 100\%$$

(5) O_2_^−^· scavenging rate

The O_2_^−^· scavenging rate was determined using the method described by Chen, K. et al. [21]. Take 200 μL of 0.05 mol/L Tris-HCl buffer (pH 8.2), preheat it in a 25 °C water bath for 20 min, add 40 μL of CP4 peptide solution at various concentrations and 40 μL of 25 mmol/L hydroquinone solution, mix well, and allow to react in a 25 °C water bath for 5 min; then, add 4 μL of 8% (*w*/*v*) HCl to stop the reaction, and measure the absorbance (*A_i_*) at a wavelength of 325 nm. Use distilled water in place of the hydroquinone solution to measure absorbance (*A_j_*); use distilled water in place of the peptide solution as a blank control to measure absorbance (*A*_0_). Use VC as a positive control and calculate the O_2_^−^· scavenging rate using the following formula:

$$O_{2^{-}}\cdot \;scavenging\;rate(\%)=1-\frac{A_{i}-A_{j}}{A_{0}}\times 100\%$$

### 2.3. Cell Experiments

#### 2.3.1. Cell Culture and Viability of HepG2 Cells

HepG2 cells were cultured in DMEM supplemented with 10% fetal bovine serum, 1% penicillin-streptomycin, and 25 mM of glucose at 37 °C in a humidified 5% CO_2_ atmosphere. For peptide safety evaluation in normal HepG2 cells, cells were exposed to CP4 at 16, 32, 64, 128, and 256 μM for 18 h, and viability was determined by the MTT assay. Because no obvious cytotoxicity was observed across this range, 32, 64, and 128 μM were selected as low, medium, and high intervention concentrations for subsequent phenotypic assays. These concentrations were chosen for in vitro biological evaluation rather than as direct estimates of concentrations achievable after oral administration.

#### 2.3.2. Establishment of the IR-HepG2 Model

The IR-HepG2 cell line was established using the method described by Ding Junfeng, with minor modifications [22]. For insulin-resistance modeling, cells were seeded in six-well plates and allowed to attach for 24 h. The control group received complete medium without glucosamine, whereas the model and CP4 treatment groups were exposed to 18 mM glucosamine prepared in serum-free high-glucose DMEM. Both 12 h and 18 h exposure conditions were tested, and the 18 h condition was selected because it produced clearer reductions in glucose consumption and glycogen content together with increased MDA and TG levels.

#### 2.3.3. Measurement of Glucose Consumption, Glycogen, TG, MDA, and ROS

After model establishment, IR-HepG2 cells were treated with CP4 (32, 64, and 128 μM) for 18 h. Glucose consumption was measured in culture supernatants. Cellular glycogen, TG, and MDA were quantified using commercial assay kits and normalized to total protein content measured by BCA. Intracellular ROS was determined using a fluorescence-based kit with excitation at 488 nm and emission at 525 nm.

#### 2.3.4. Cellular Localization of FITC-Labelled CP4

An IR-HepG2 model was established as described above, after which cells were incubated with FITC-labelled CP4 (64 μM) for 18 h. ActinRed and DAPI were used to label the cytoskeleton and nuclei, respectively, and fluorescence images were obtained using a Zeiss laser scanning confocal microscope.

### 2.4. Transcriptomics

Total RNA was isolated from control, model, and peptide-treated cohorts (*n* = 3) employing Trizol reagent (Takara, Kusatsu, Japan). RNA concentration and purity were assessed via Nanodrop2000 spectrophotometry, integrity verified by agarose gel electrophoresis, and RNA quality number (RQN) values determined using Agilent5300 (Agilent, Santa Clara, CA, USA). Eukaryotic mRNA possessing 3′-terminal polyadenylation underwent oligodT-mediated magnetic bead capture for selective isolation from total RNA. Fragmentation buffer introduction generated randomized ~300 bp mRNA fragments. Reverse transcription utilized random hexamers to synthesize first-strand cDNA from fragmented mRNA templates, followed by double-stranded cDNA synthesis. Cohesive termini of dsDNA were blunt-ended using End Repair Mix, then 3′-adenylated for adapter ligation. Adapter-ligated constructs were size-selected and PCR-amplified to generate sequencing libraries. NovaSeq X Plus platform sequencing produced raw data, with gene expression quantitation derived from genomic region-aligned clean reads (RSEM v1.3.3 software, https://deweylab.github.io/RSEM/). Differential expression analysis (limma package) identified significantly altered transcripts (*p* < 0.05 & |log2FC| ≥ 0.585) across ≥2 sample groups.

### 2.5. Metabolomics

Aliquot 100 μL of sample into a 1.5 mL microcentrifuge tube, supplement with 400 μL of extraction solvent (acetonitrile:methanol, 1:1 *v*/*v*) spiked with 0.02 mg/mL L-2-chlorophenylalanine internal standard. Vortex vigorously for 30 s, then subject to cryogenic ultrasonication (5 °C, 40 kHz, 30 min). Incubate at −20 °C for 30 min, perform centrifugation (4 °C, 13,000× *g*, 15 min), and decant supernatant. Concentrate under N_2_ stream, reconstitute in 100 μL of dissolution solvent (acetonitrile:water, 1:1 *v*/*v*), perform ultrasonication (5 °C, 40 kHz, 5 min), then perform recentrifugation (4 °C, 13,000× *g*, 10 min). Transfer clarified supernatant to an LC vial with insert for UHPLC-Q Exactive HF-X analysis (Thermo Fisher Scientific, Waltham, MA, USA). Chromatographic separation employed an HSS T3 column (100 × 2.1 mm, 1.8 μm) with 3 μL injection. Mobile phases comprised: (A) 0.1% formic acid in H_2_O:ACN (95:5 *v*/*v*); (B) 0.1% formic acid in ACN:IPA:H_2_O (47.5:47.5:5 *v*/*v*). Conditions were 0.40 mL/min flow rate and 40 °C column temperature. Mass spectrometric detection utilized positive/negative polarity switching (*m*/*z* 70–1050) with sheath gas (50 psi), auxiliary gas (13 psi, 425 °C), spray voltages (±3500 V), and ion transfer tube (325 °C). MS^1^ resolution: 60,000; MS^2^: 7500 in data-dependent acquisition mode with stepped normalized collision energy (20/40/60 eV).

### 2.6. Integrated Analysis of Transcriptomics and Metabolomics

Using the Metscape plugin in Cytoscape v3.10.4, we integrated overlapping targets, differentially expressed genes, and differentially expressed metabolites, and constructed a compound-reaction-enzyme-gene interaction framework for a holistic interpretation of metabolic pathways.

### 2.7. Real-Time Quantitative PCR

HepG2 cells were treated as described in Section 2.3.3. Total RNA was then extracted from the cells using Trizol reagent and diluted to 500 ng/mL. After removing genomic DNA using a reverse transcription kit, the total RNA was reverse-transcribed into cDNA. The PCR reaction mixture contains cDNA, primers, RNase-free water, and SYBR Green qPCR master mix. The primer pair sequences are shown in Table 2. The reaction conditions are as follows: 3 min at 95 °C, followed by 40 cycles, each consisting of 5 s at 95 °C and 34 s at 60 °C.

### 2.8. Data Analysis

In this study, with the exception of the transcriptomic and metabolomic data, which were processed using the Meiji Cloud platform (https://www.majorbio.com/) and the dedicated software Cytoscape 3.10.4, all other experimental data were analyzed statistically and plotted using GraphPad Prism 8.0 software. Results are presented as mean ± standard deviation (SD). Two-group comparisons were performed using Student’s *t*-test, while multi-group comparisons were performed using one-way ANOVA; a value of *p* < 0.05 was considered statistically significant.

## 3. Results

### 3.1. In Vitro Characterization of CP4

#### 3.1.1. Hemolytic Properties of CP4

**Figure 1:**
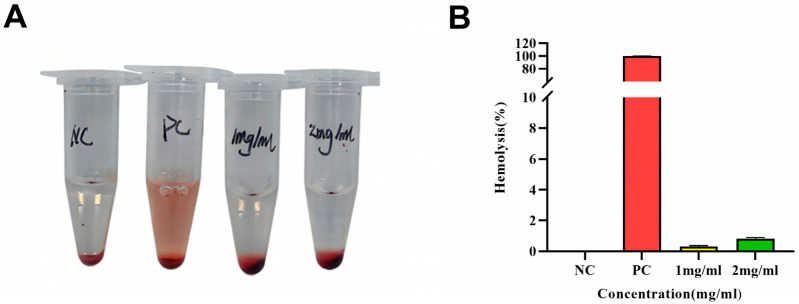
Hemolytic activity of CP4. (**A**) Hemolytic activity of CP4 against mice red blood cells. (**B**) Hemolytic activity assay. NC: negative control (PBS pH 7.4); PC: positive control (0.1% Triton X-100).

In vitro hemolysis experiments were conducted to evaluate the hemolytic risk of CP4 on mammalian red blood cells and to ensure its hemocompatibility and preliminary safety in living organisms. As shown in Figure 1, at concentrations ranging from 1 mg/mL to 2 mg/mL, the hemolysis rates of CP4 were 0.31% and 0.81%, respectively, with both rates remaining below 5% for mammalian red blood cells. When the hemolysis rate induced by a biomaterial is below 5%, it is considered to be hemocompatible [23]. Good hemocompatibility helps prevent adverse reactions such as hemolysis, thrombosis, or platelet activation. Therefore, CP4 does not cause the lysis of mammalian red blood cells within this concentration range and demonstrates good preliminary safety.

#### 3.1.2. Gastrointestinal Stability of CP4

**Figure 2:**
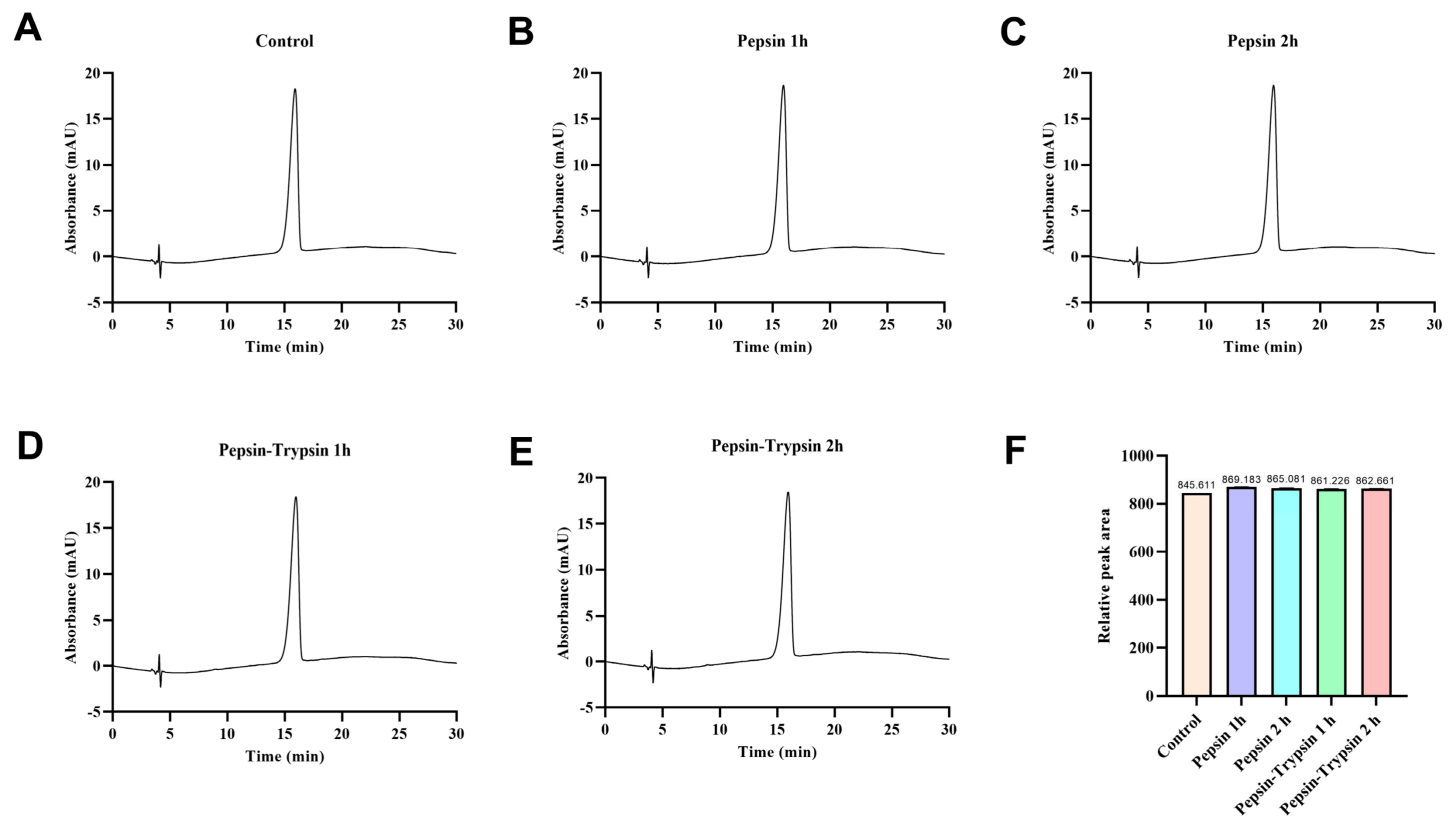
CP4 In Vitro Gastrointestinal Stability Analysis. (**A**) Control group. (**B**) Pepsin 1 h. (**C**) Pepsin 2 h. (**D**) Trypsin 1 h. (**E**) Trypsin 2 h. (**F**) Peak area.

As a peptide molecule, CP4 must first be able to withstand gastrointestinal digestion in order to exert its biological effects in vivo. In this study, in vitro experiments simulating gastrointestinal digestion were conducted, and RP-HPLC was used to detect changes in CP4 following treatment with pepsin and trypsin. As shown in Figure 2, the HPLC profile of CP4 remained largely unchanged after 2 h of pepsin digestion, with no new characteristic peaks or significant alterations in peak shape observed. Following this, trypsin digestion was performed; after 1 and 2 h of treatment, the retention times and shapes of the main peaks remained consistent. Calculation of peak areas (as shown in Figure 2F) revealed that the retention times of the main peak of CP4 remained essentially consistent after treatment with both pepsin and trypsin, and no significant decrease in peak area was observed. These findings suggest that CP4 possesses favorable gastrointestinal stability under simulated digestion conditions.

#### 3.1.3. Inhibitory Effect of CP4 on α-Glucosidase

**Figure 3:**
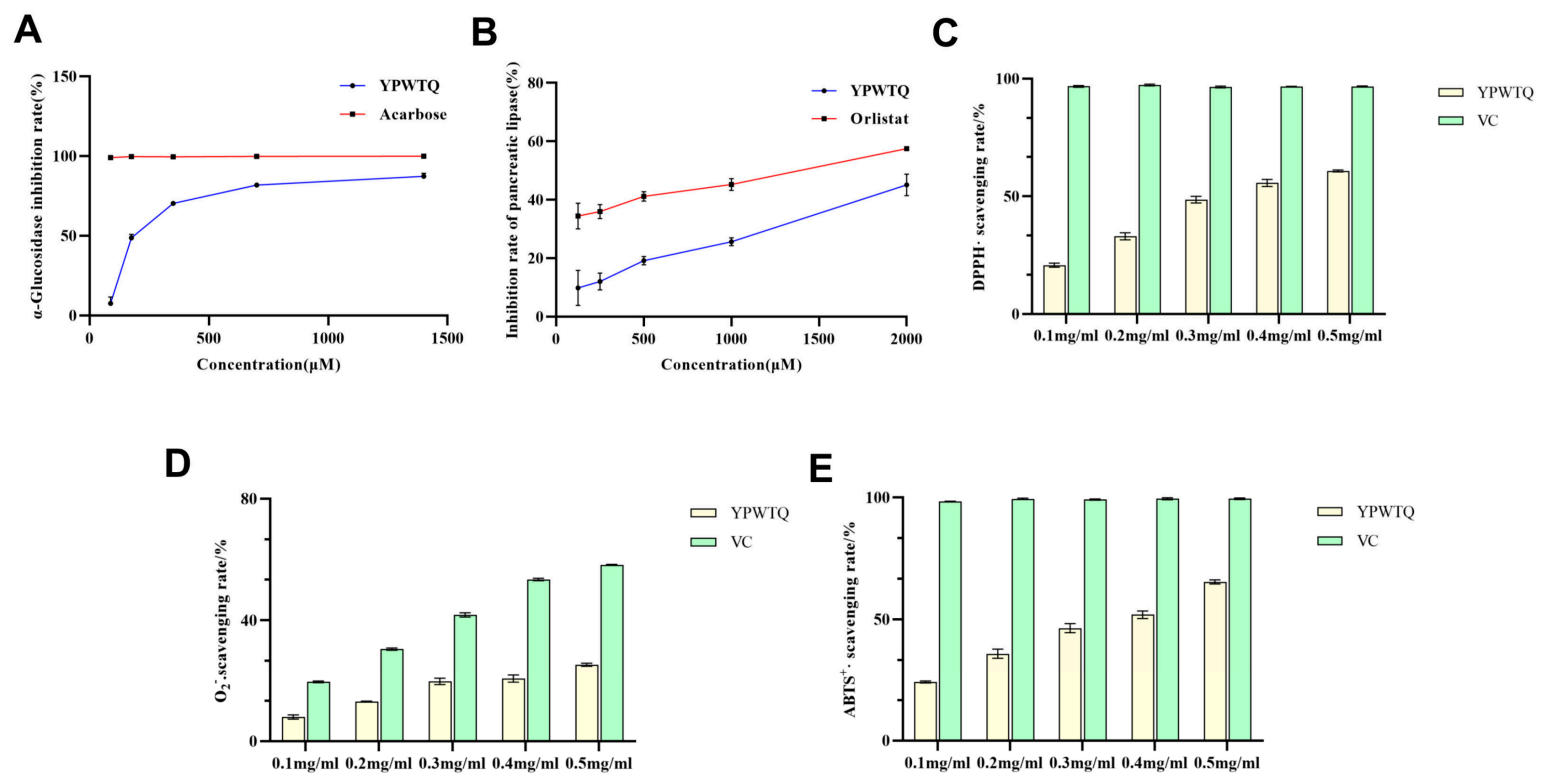
CP4’s (**A**) α-glucosidase inhibition rate; (**B**) pancreatic lipase inhibition rate; (**C**) DPPH radical scavenging rate; (**D**) superoxide anion radical scavenging rate; and (**E**) ABTS cation radical scavenging rate.

Given the general characteristic of food-derived hypoglycemic peptides to exert synergistic hypoglycemic effects through multiple targets, and to comprehensively elucidate the hypoglycemic mechanism of CP4, we further selected α-glucosidase—another key hypoglycemic target in T2DM—and investigated the inhibitory effect of CP4 on this target through in vitro enzyme activity assays, thereby systematically elucidating the multi-target hypoglycemic activity of CP4 [24]. Figure 3A shows that CP4 exhibits good inhibitory activity against α-glucosidase. At a CP4 concentration of 1400 μM, the α-glucosidase inhibition rate reaches 87.26%. Previous studies have shown that potential α-glucosidase inhibitory peptides are typically rich in hydrophobic amino acid residues [25], and the CP4 sequence contains highly hydrophobic amino acids such as Tyr, Pro, and Trp, which may be related to its strong α-glucosidase inhibitory activity.

#### 3.1.4. Inhibitory Effect of CP4 on Pancreatic Lipase

As the central enzyme responsible for breaking down dietary triglycerides (TG) in the intestine, pancreatic lipase plays a key role in lipid digestion and absorption by catalyzing the hydrolysis of TG into free fatty acids and monoglycerides. Therefore, inhibiting the activity of this enzyme has become an important strategy for intervening in obesity and related lipid metabolism disorders [26]. Figure 3B indicates that CP4 exhibited moderate inhibitory activity against pancreatic lipase in a concentration-dependent manner. At a concentration of 2000 μM, the inhibition rate of Orlistat was approximately 57.51%, while that of CP4 was about 45.08%. Although the inhibitory effect of CP4 on pancreatic lipase is slightly less potent than that of the clinically used drug Orlistat, it still demonstrates good inhibitory potential.

#### 3.1.5. The Antioxidant Activity of CP4

CP4 also exhibits good antioxidant activity. At a concentration of 0.5 mg/mL, its scavenging rates for DPPH·, ABTS^+^·, and O_2_^−^· reached 60.82%, 65.35%, and 25.40%, respectively (Figure 3C–E).

### 3.2. Cell Experiments

#### 3.2.1. Selection of Active Peptide Concentration

**Figure 4:**
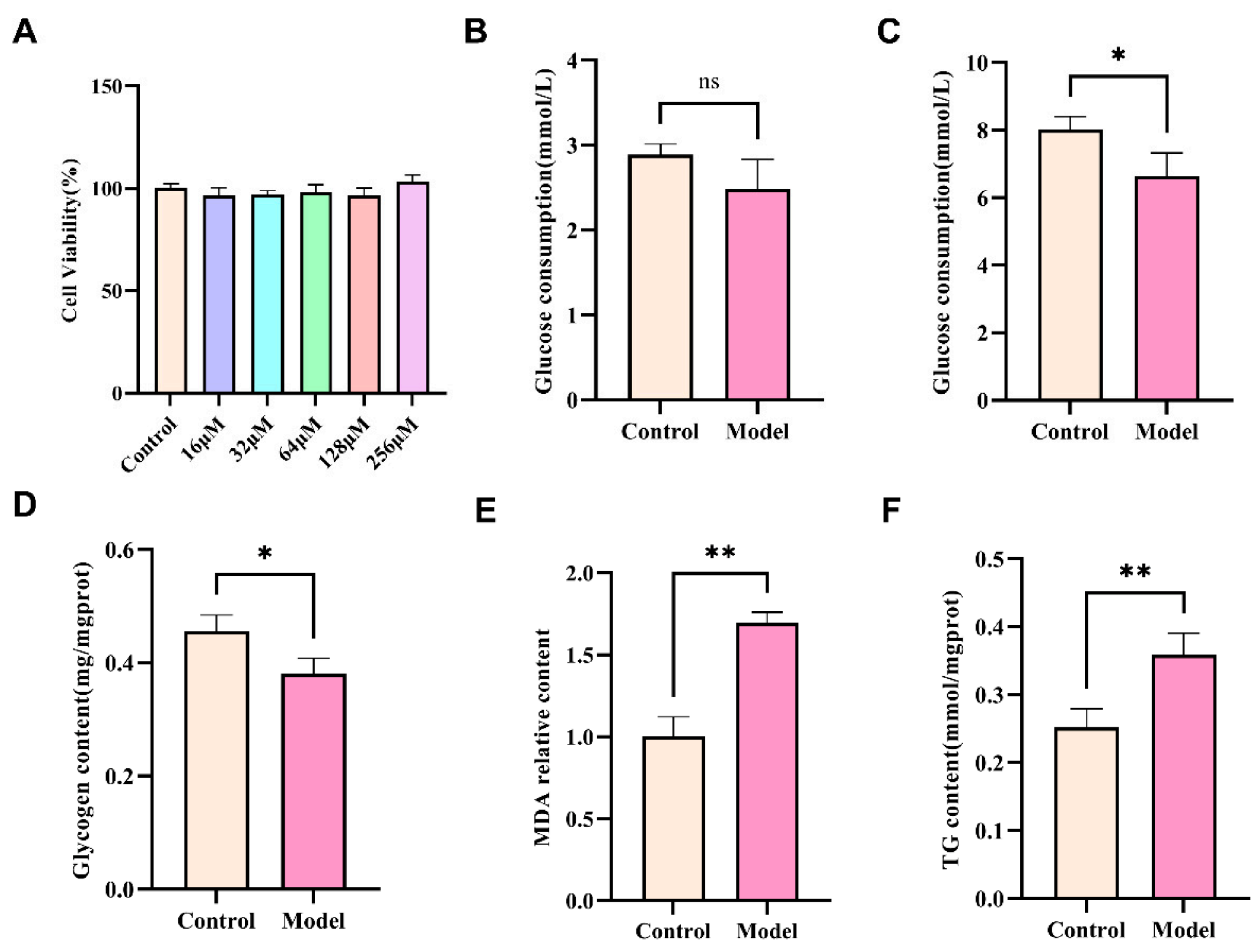
(**A**) Cell viability. (**B**) Effect of 18 mM glucosamine treatment for 12 h on glucose consumption in HepG2 cells. (**C**) Effect of 18 mM glucosamine treatment for 18 h on glucose consumption HepG2 cells. (**D**) Glycogen content. (**E**) MDA content. (**F**) TG content. * *p* < 0.05; ** *p* < 0.01.

According to previous reports, if cell viability exceeds 80% after peptide treatment, the peptide is generally considered non-cytotoxic [27]. As presented in Figure 4A, CP4 exhibits no statistically significant deviation from the control group across concentrations spanning 16 to 256 μM. Therefore, 32, 64, and 128 μM were selected as low, medium, and high concentration groups for subsequent assays.

#### 3.2.2. Establishment of IR-HepG2 Cell Model

Insulin resistance (IR) is one of the typical manifestations of T2DM [28]. To evaluate the therapeutic potential of CP4 for T2DM, a glucosamine-induced insulin-resistant HepG2 cell model was established and assessed using biomarkers such as glucose consumption, glycogen, MDA, and TG levels. Figure 4B shows that there was no significant difference in glucose consumption between the model group and the control group after 12 h of exposure to 18 mM glucosamine (*p* > 0.05). However, 18 h of treatment significantly inhibited cellular glucose consumption (Figure 4C). Compared with the control group, IR-HepG2 cells exhibited significantly reduced glycogen synthesis capacity (Figure 4D), as well as significantly elevated MDA (Figure 4E) and TG (Figure 4F) levels. Collectively, these findings confirm that sustained 18 mM glucosamine treatment for 18 h induces insulin resistance in HepG2 cells by impairing glucose consumption, depleting glycogen reserves, and increasing MDA and TG accumulation.

#### 3.2.3. Cytotoxic Evaluation of CP4 in Insulin-Resistant HepG2 Cells

**Figure 5:**
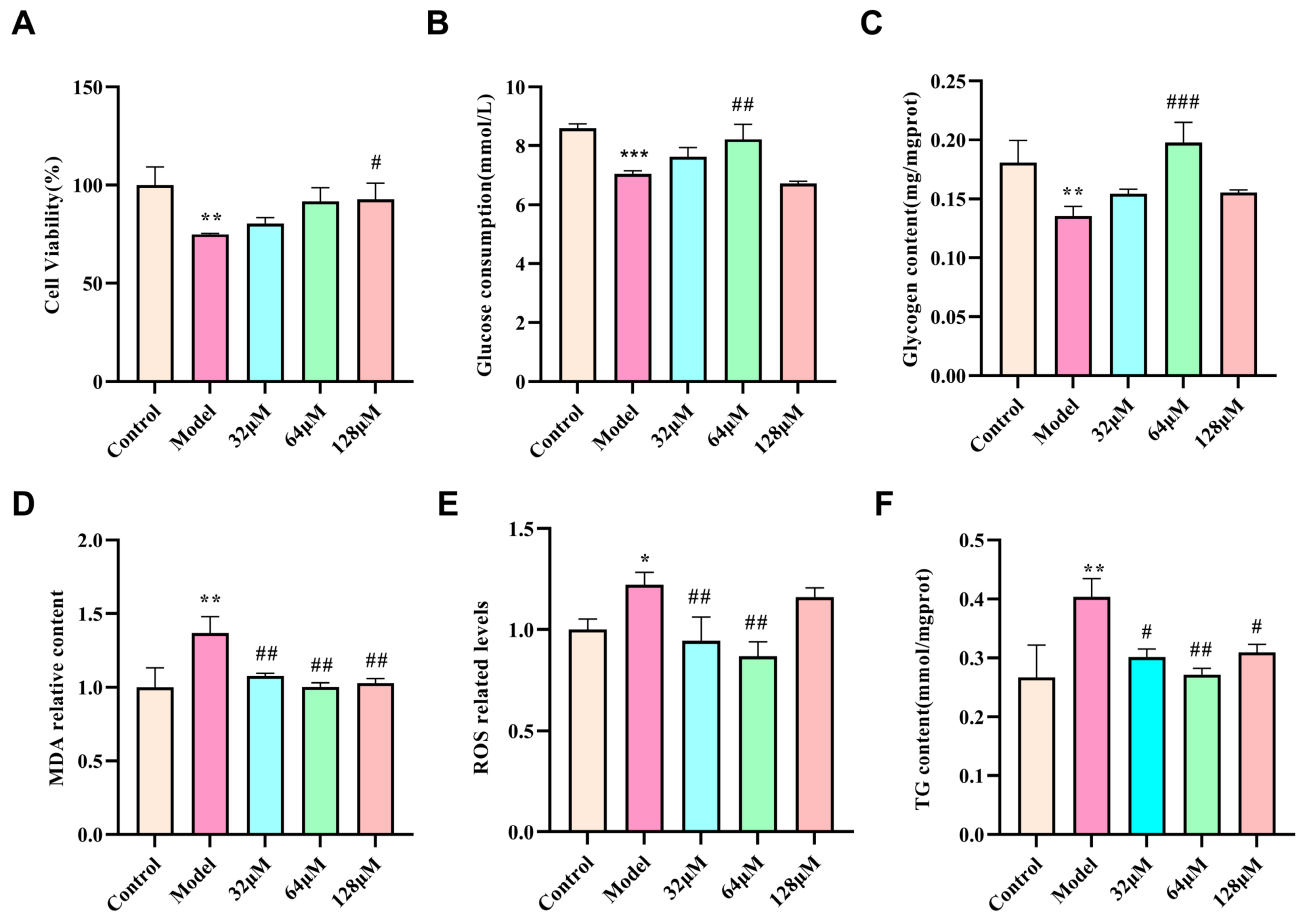
Effects of CP4 on IR-HepG2 (**A**) Cell viability; (**B**) glucose consumption; (**C**) glycogen content; (**D**) MDA content; (**E**) ROS content; and (**F**) TG content. * *p* < 0.05, ** *p* < 0.01, and *** *p* < 0.001: compared with the control group. ^#^ *p* < 0.05, ^##^ *p* < 0.01, and ^###^ *p* < 0.001: compared with the model group.

As shown in Figure 5A, the results indicate that after 18 h of treatment with 18 mM of glucosamine, the cell survival rate of HepG2 cells decreased to 75.00% of the control group. CP4 treatment dose-dependently improved the viability of IR-HepG2 cells.

#### 3.2.4. Effect of CP4 on Glucose Consumption in Cells

Because impaired cellular glucose uptake is a hallmark of insulin resistance, glucose consumption was used as a primary indicator of CP4 activity. Model group HepG2 cells were treated with different concentrations of CP4 for 18 h. Figure 5B shows that in the IR-HepG2 cells, the peptide substance enhanced glucose consumption, with 64 μM CP4 significantly increasing glucose consumption compared to the Model group (*p* < 0.01).

#### 3.2.5. Effect of CP4 on Glycogen Content in Cells

Glycogen, a branched glucose polymer functioning as a physiological energy reservoir, exhibits primary deposition within skeletal musculature and hepatic tissue. Therefore, glycogen synthesis and gluconeogenesis are key regulatory mechanisms controlling glucose release from the liver [29]. Figure 5C shows that glycogen content was significantly reduced in the model group compared to the control group (*p* < 0.01), confirming that glycogen synthesis was inhibited in the glucosamine-induced model group. Compared to the model group, administration of 64 μM and 128 μM of CP4 resulted in a significant increase in glycogen content, indicating that CP4 can stimulate glycogen synthesis while improving insulin resistance.

#### 3.2.6. Effect of CP4 on MDA and ROS Content in Cells

Oxidative stress is a major pathogenic factor in the pathogenesis of IR [30]. Inhibiting oxidative stress can serve as an adjunctive therapeutic strategy for glycemic control and alleviation of diabetic complications [31]. In this study, the efficacy of CP4 in alleviating oxidative stress in the IR-HepG2 model system was quantitatively assessed by measuring MDA and ROS levels. Figure 5D,E show that, compared with the control group, MDA and ROS levels were significantly elevated in IR-HepG2 cells (*p* < 0.01), confirming that IR leads to impaired cellular antioxidant defense functions. Compared with the insulin resistance group, CP4 administration (64 μM) significantly reduced the levels of both oxidative stress markers, indicating that this peptide can induce the restoration of redox homeostasis and provide cells with protective effects against oxidative damage.

#### 3.2.7. Effect of CP4 on TG Content in Cells

Excessive lipid accumulation is a prerequisite for obesity and the development of diabetes [32]. The experiment assessed the effect of CP4 on lipid metabolism in IR-HepG2 cells by measuring TG. As shown in Figure 5F, the TG content in IR-HepG2 cells was significantly higher than that in control group, indicating that insulin resistance promotes lipid accumulation in cells. Compared with the IR-HepG2 group, the TG content in the CP4 group was significantly reduced. This suggests that CP4 can inhibit the accumulation of TG in IR-HepG2 cells and regulate cellular lipid metabolism.

#### 3.2.8. Dynamic Observation of the Effect of CP4 on HepG2 Cells

**Figure 6:**
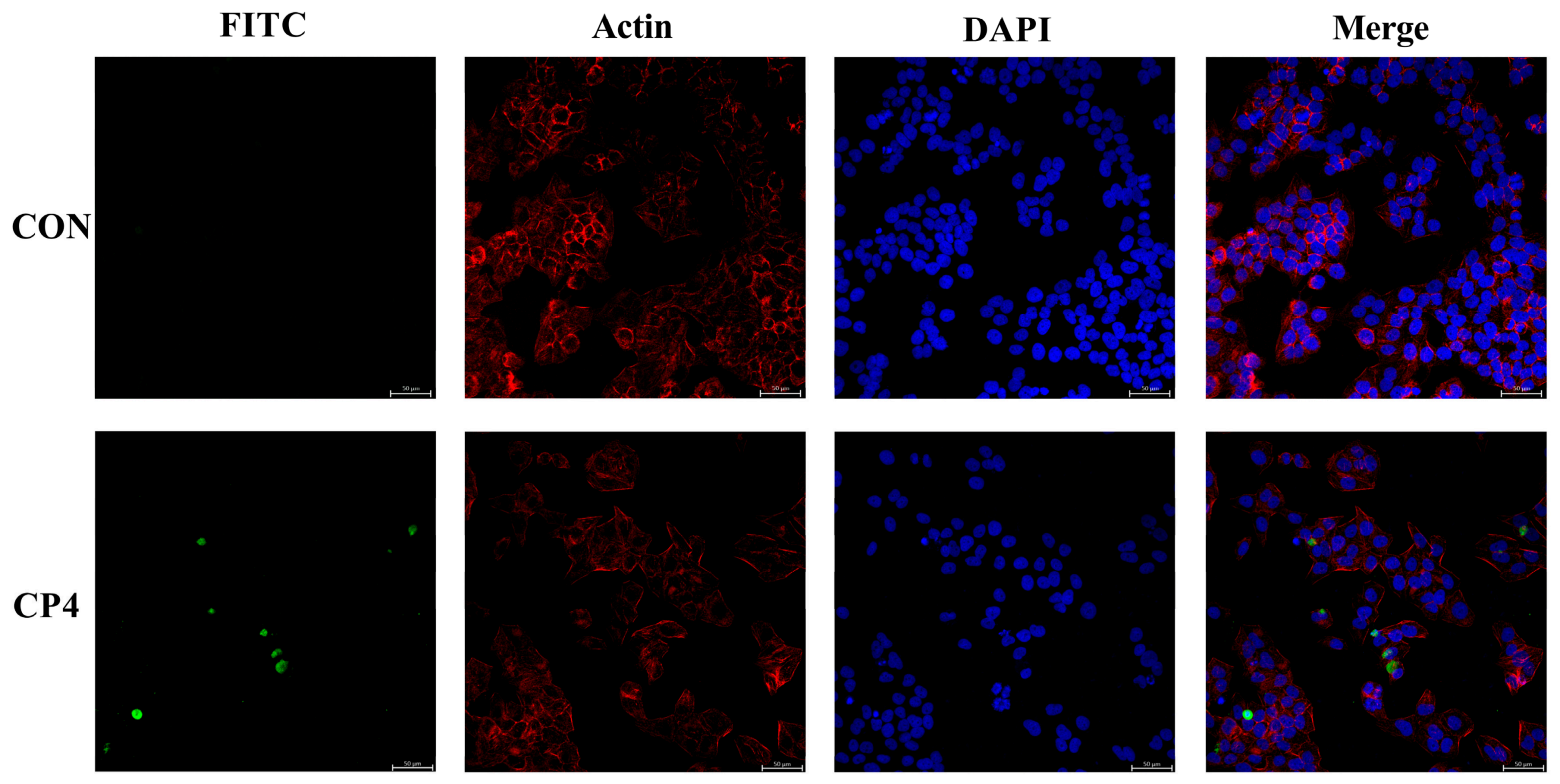
Fluorescence image of fluorescently labeled CP4 (HepG2 cell nuclei and structures labeled with DAPI (blue) and actin (red)).

To investigate whether CP4 can cross the cell membrane, HepG2 nuclei were counterstained with DAPI (blue), cytoskeletal architecture with actin (red), and the peptide with FITC (green). As shown in Figure 6, green fluorescence was detected within the cytoplasm of HepG2 cells. This indicates that FITC-labeled CP4 can cross the cell membrane and enter HepG2 cells.

### 3.3. Transcriptomics

**Figure 7:**
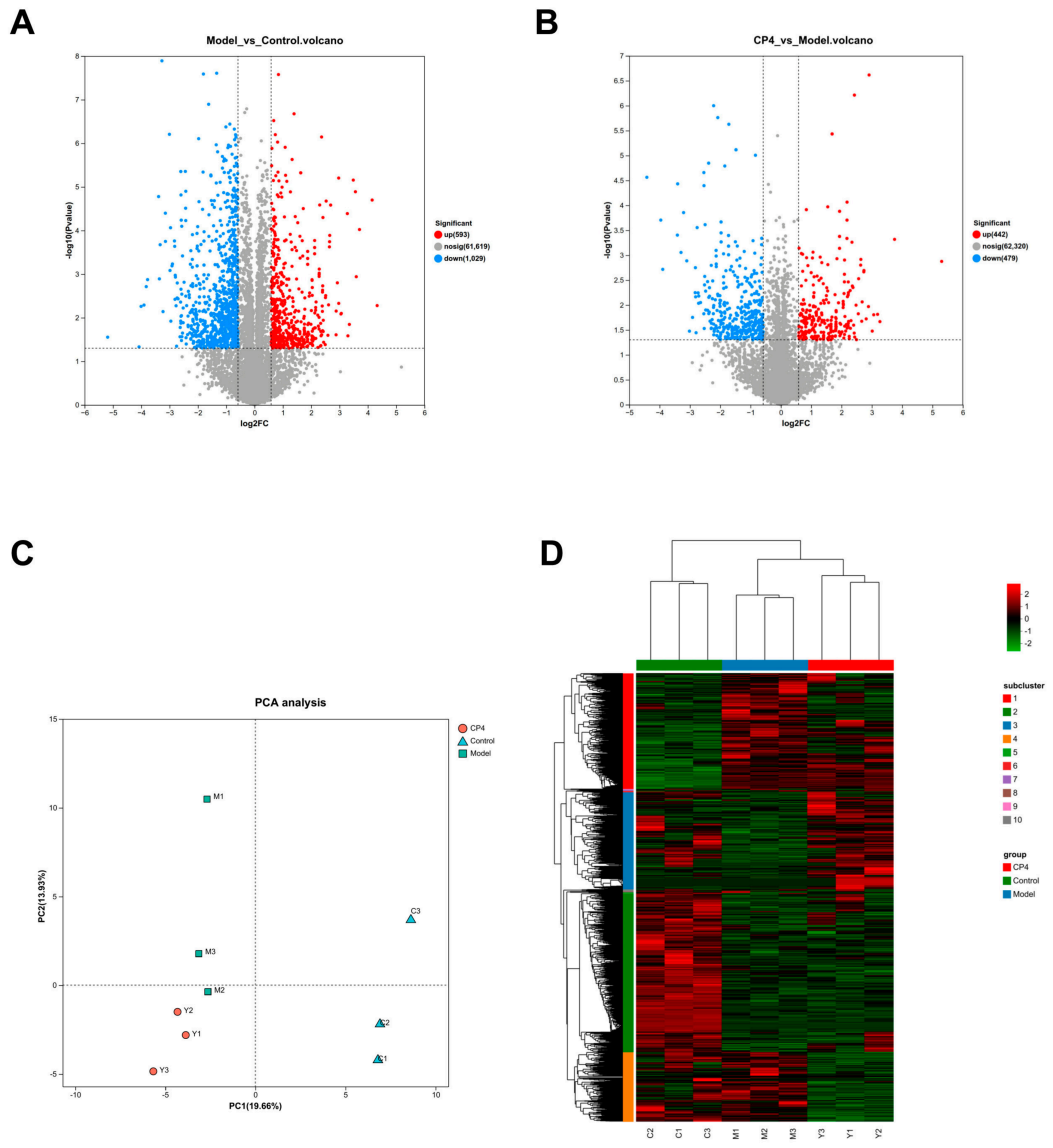
(**A**) Volcano plot of upregulated and downregulated DEGs between the model and control groups. (**B**) Volcano plot of upregulated and downregulated DEGs between the CP4 and model groups. (**C**) PCA plot of samples from the control, model, and CP4 treatments. (**D**) Heatmap of hierarchical clustering analysis of DEGs among samples.

To delineate T2DM therapeutic targets, transcriptomic profiling of control, model, and CP4-treated cohorts was conducted via Illumina high-throughput sequencing. Nine specimens yielded 69.05 Gb of high-fidelity reads (≥6.74 Gb/sample, Q30 > 96.22%). Comparative analysis revealed 1622 transcriptional alterations in model versus control cohorts (593 upregulated, 1029 downregulated; Figure 7A), while CP4 intervention elicited 921 differentially expressed genes relative to model systems (442 upregulated, 479 downregulated; Figure 7B). Principal component analysis (PCA) demonstrated discernible intergroup segregation (Figure 7C), confirming model establishment and CP4 intervention efficacy. Clustered differential expression pattern analysis was performed to visualise expression trends across groups, as depicted in Figure 7D. Each column in the figure represents a sample, and each row represents a gene. The colors in the figure indicate the expression level of the gene/transcript in that sample. By default, red indicates a high expression level of the gene/transcript in that sample, while blue indicates a low expression level. For specific trends in expression levels, please refer to the numerical labels below the color bar in the upper right corner. On the left is a clustering dendrogram for genes/transcripts; the closer two gene/transcript branches are to each other, the more similar their expression levels are. At the top is a clustering dendrogram for samples, and below are the sample names; the closer two sample branches are to each other, the more similar the expression patterns of all genes/transcripts in those two samples are—that is, the more similar the trends in gene/transcript expression levels are.

**Figure 8:**
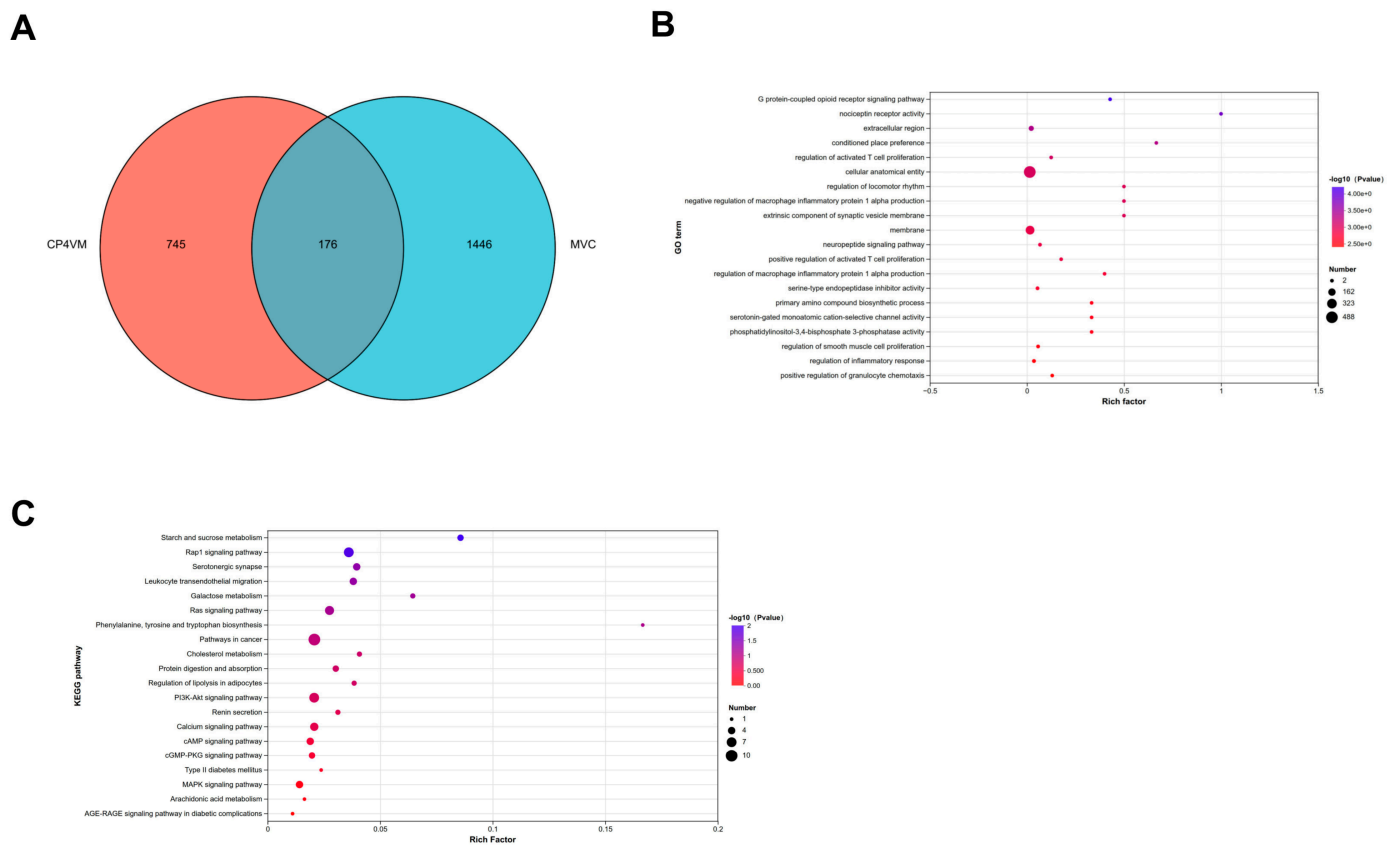
(**A**) Venn diagram of differentially expressed genes in the Model, Control, and CP4 groups. GO (**B**) and KEGG (**C**) analyses of differentially expressed genes in the CP4 and model groups.

Venn diagram analysis identified 176 common targets between the model group and control group, and between the CP4-treated group and model group (Figure 8A). GO enrichment analysis (Figure 8B) revealed that, compared to the model group, differentially expressed genes in the CP4-treated group were primarily associated with G protein-coupled receptor signalling pathways, downregulation of macrophage inflammatory protein 1α, neuropeptide signalling pathways, extracellular regions, cellular anatomical structures, synaptic vesicle membrane external components, and cell membranes. KEGG pathway analysis (Figure 8C) indicated that, compared to the model group, CP4-treated cells exhibited transcriptional alterations in starch and sucrose metabolism, the Rap1 signalling pathway, the Ras signalling pathway, cholesterol metabolism, regulation of lipolysis in adipocytes, the PI3K-Akt signalling pathway, the cAMP signalling pathway, type 2 diabetes, the MAPK signalling pathway, the AGE-RAGE signalling pathway in diabetic complications, and arachidonic acid metabolism. For details on the expression profiles of differentially expressed genes, see Table S1.

### 3.4. Metabolomics

**Figure 9:**
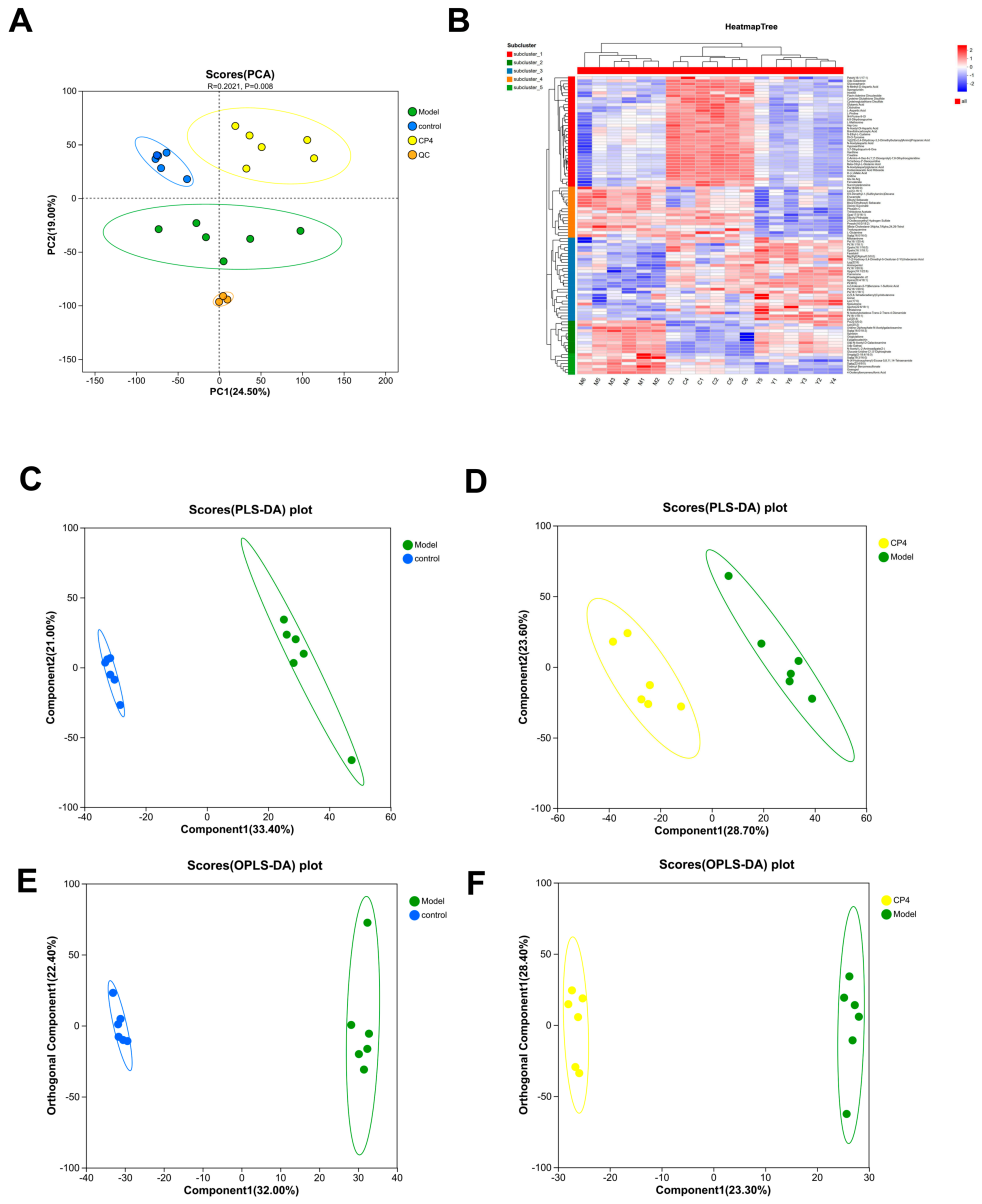
(**A**) PLS-DA score plots for control vs. model and model vs. CP4. (**B**) Heatmap of differentially expressed metabolites. (**C**) PLS-DA between the control group and the model group. (**D**) PLS-DA between the model group and the CP4 group. (**E**) OPLS-DA between the control group and the model group. (**F**) OPLS-DA between the model group and the CP4 group.

Multivariate interrogation of metabolomic profiles delineated systemic metabolic alterations across cohorts. PCA revealed distinct clustering tendencies among experimental groups (Figure 9A). Differential metabolite screening employed a discriminatory threshold (*p* < 0.05, VIP > 1.0). Thermographic representation of global metabolite expression patterns demonstrated clustering relationships and restoration of metabolic homeostasis following CP4 intervention, indicating attenuated metabolic perturbation (Figure 9B). Partial least squares–discriminant analysis (PLS-DA) exhibited significant segregation between control and CP4 cohorts relative to model systems (Figure 9C,D). Orthogonal PLS-DA (Figure 9E,F) confirmed robust separability in control–model and model–CP4 comparisons.

**Figure 10:**
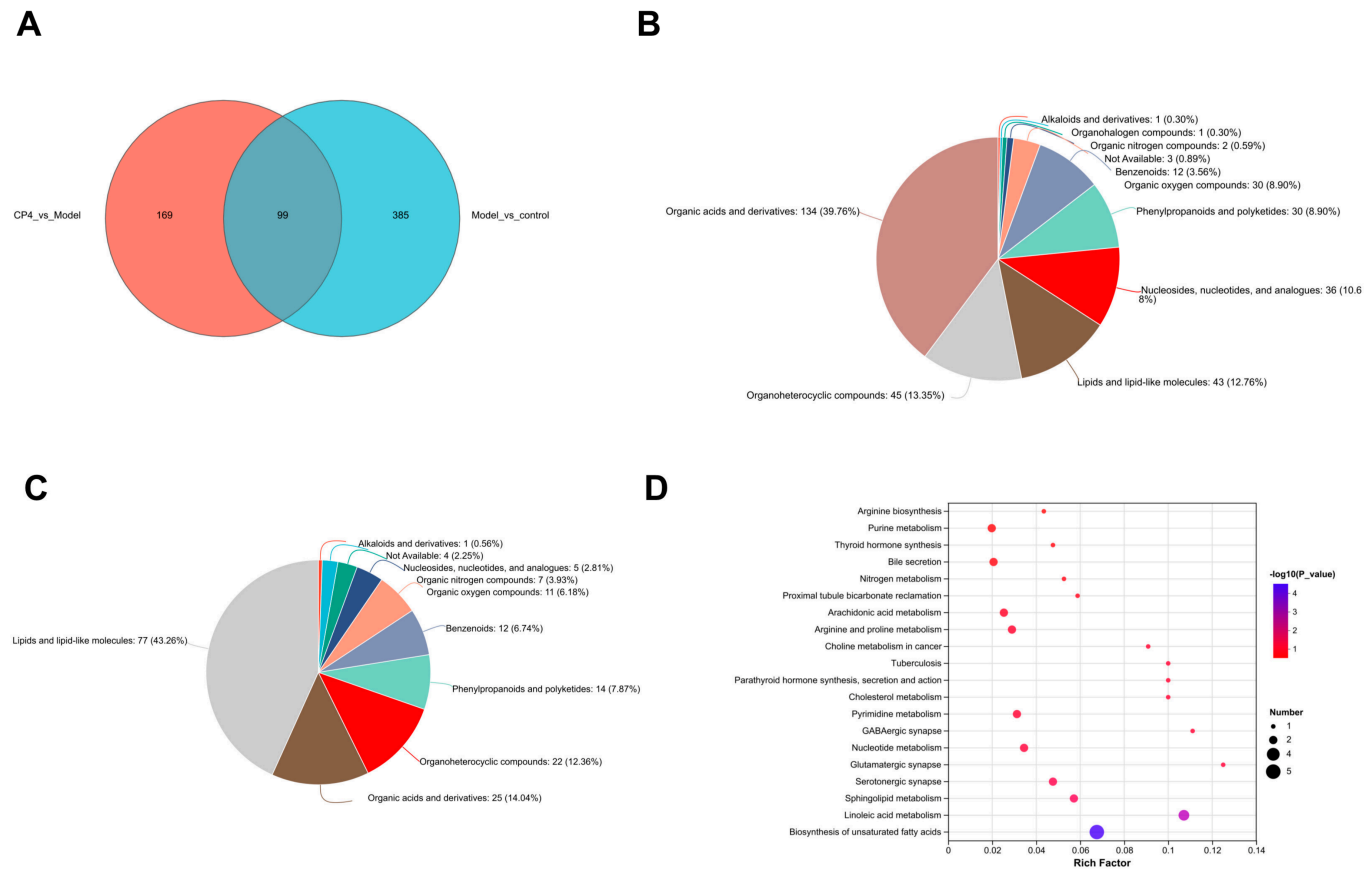
(**A**) Venn diagram of differential metabolites between the model group, control group, and CP4 group. (**B**) Classification diagram of differential metabolites between the model group and the control group. (**C**) Classification diagram of differential metabolites between the treatment group and the model group. (**D**) Metabolic pathways of important metabolites in model vs. CP4.

Venn analysis revealed 99 cross-metabolites between the model control group and the CP4-treated group (Figure 10A). To identify the metabolite categories affected by CP4 intervention, this study classified the screened differential metabolites. Results in Figure 10B indicate that differential metabolites between the model and control groups were primarily enriched in organic acids and their derivatives (39.76%), organic heterocyclic compounds (13.35%), and lipids and lipoid molecules (12.76%). Among these, organic acids and their derivatives accounted for the highest proportion, suggesting that the occurrence of insulin resistance is closely related to disturbances in energy metabolism and lipid metabolism. Figure 10C shows that the proportion distribution of differential metabolites between the model group and the CP4 group underwent significant changes. Lipids and lipoid molecules rose to the top position (43.26%), followed by organic acids and their derivatives (14.04%) and organic heterocyclic compounds (12.36%). Comparing the classification results of the two groups reveals that CP4 hypoglycemic peptides primarily improve metabolic disorders in diabetes by regulating lipid metabolism and organic acid metabolic pathways. This finding provides a clear metabolic direction for subsequent elucidation of the mechanism of action of CP4. KEGG enrichment analysis revealed pathways involving CP4-regulated metabolites (Figure 10D), including purine metabolism, bile secretion, arachidonic acid metabolism, cholesterol metabolism, serotonergic synapse, sphingomyelin metabolism, linoleic acid metabolism, and unsaturated fatty acid biosynthesis. Significantly CP4-regulated metabolites included 4-(glutaminyl)butanoic acid, prostaglandin J2, γ-linolenic acid, linoleic acid, 12,13-dihydroxyoleic acid, and cinnamic acid, as detailed in Table S2.

### 3.5. Multi-Omics Analysis

**Figure 11:**
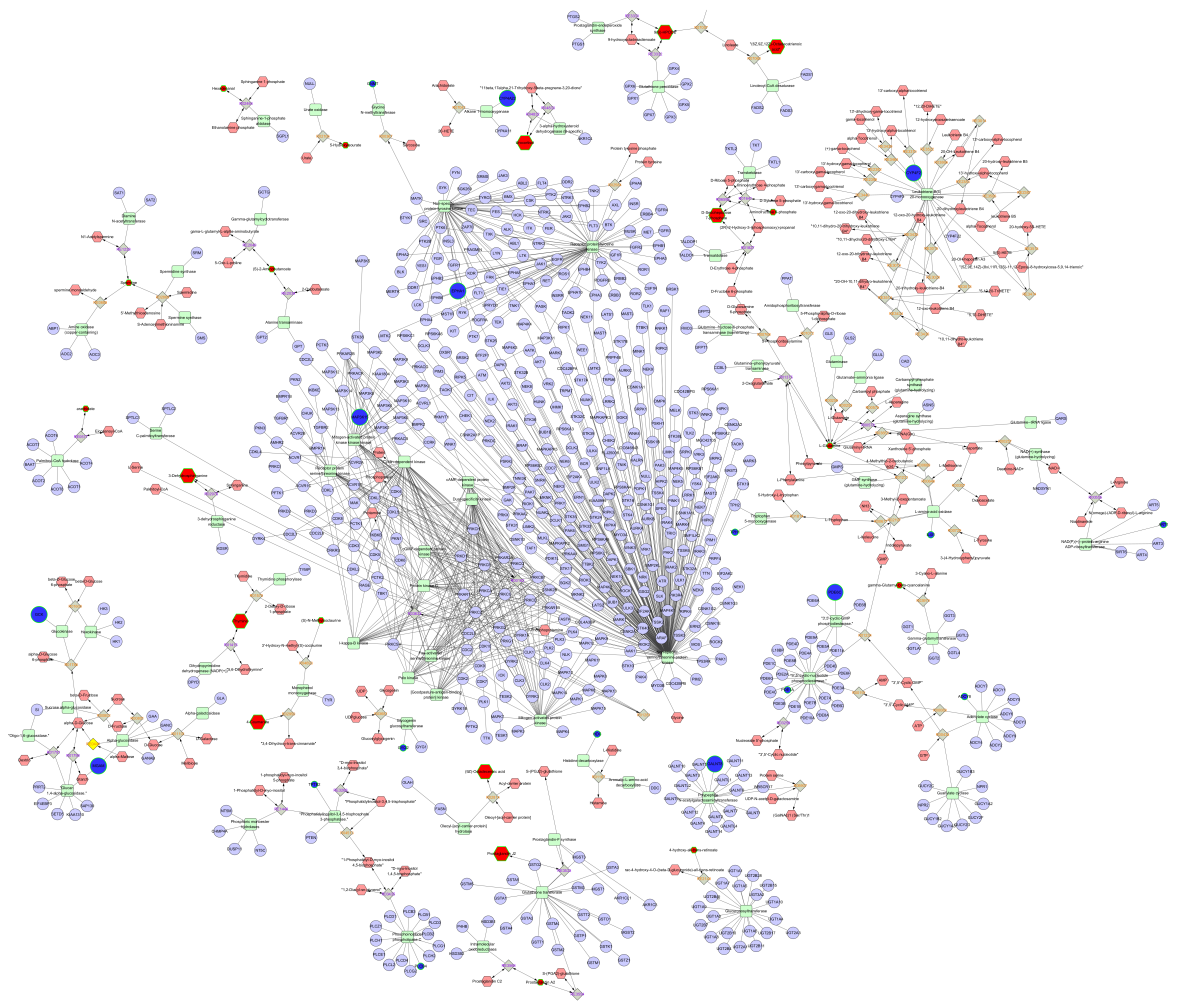
Metabolite–enzyme–reaction–gene network diagram.

To comprehensively elucidate the anti-diabetic mechanism of CP4, an integrative regulatory network was established through the convergence of network pharmacology, transcriptomic (RNA-seq), and metabolomic profiling (Figure 11). Detected differentially expressed genes and metabolites were incorporated into the MetScape plugin to generate a compound–reaction–enzyme–gene interaction framework. Subsequent integration of transcriptional alterations with compound data revealed 18 pivotal molecular targets (blue circles in Figure 10), including EPHA1, MAP3K15, TPH1, IL4I1, ART1, PDE6C, PDE1A, ADCY8, GALNT8, CYP4A22, GNMT, CYG2, HDC, CYP4F2, GCK, MGAM, TPTE2, and PLCB4, as well as the associated key metabolites 6-phosphate aminofructose, D-7-phosphate sedoheptulose, L-glutamine, 5-hydroxyisoleucine, spermine, 3-dehydro-dihydro-sphinganine,γ-glutamyl-β-cyanoalanine, thymine, (S)-N-Methylcoclaurine, Prostaglandin A2, and Prostaglandin J2 (red hexagons in Figure 10). Metabolic pathways were prioritized based on their enrichment significance and known associations with glycolipid metabolism, oxidative stress, and diabetes complications. Consequently, the PI3K-Akt, Rap1, AGE-RAGE, Ras, and related metabolic pathways were identified as candidate pathways.

**Figure 12:**
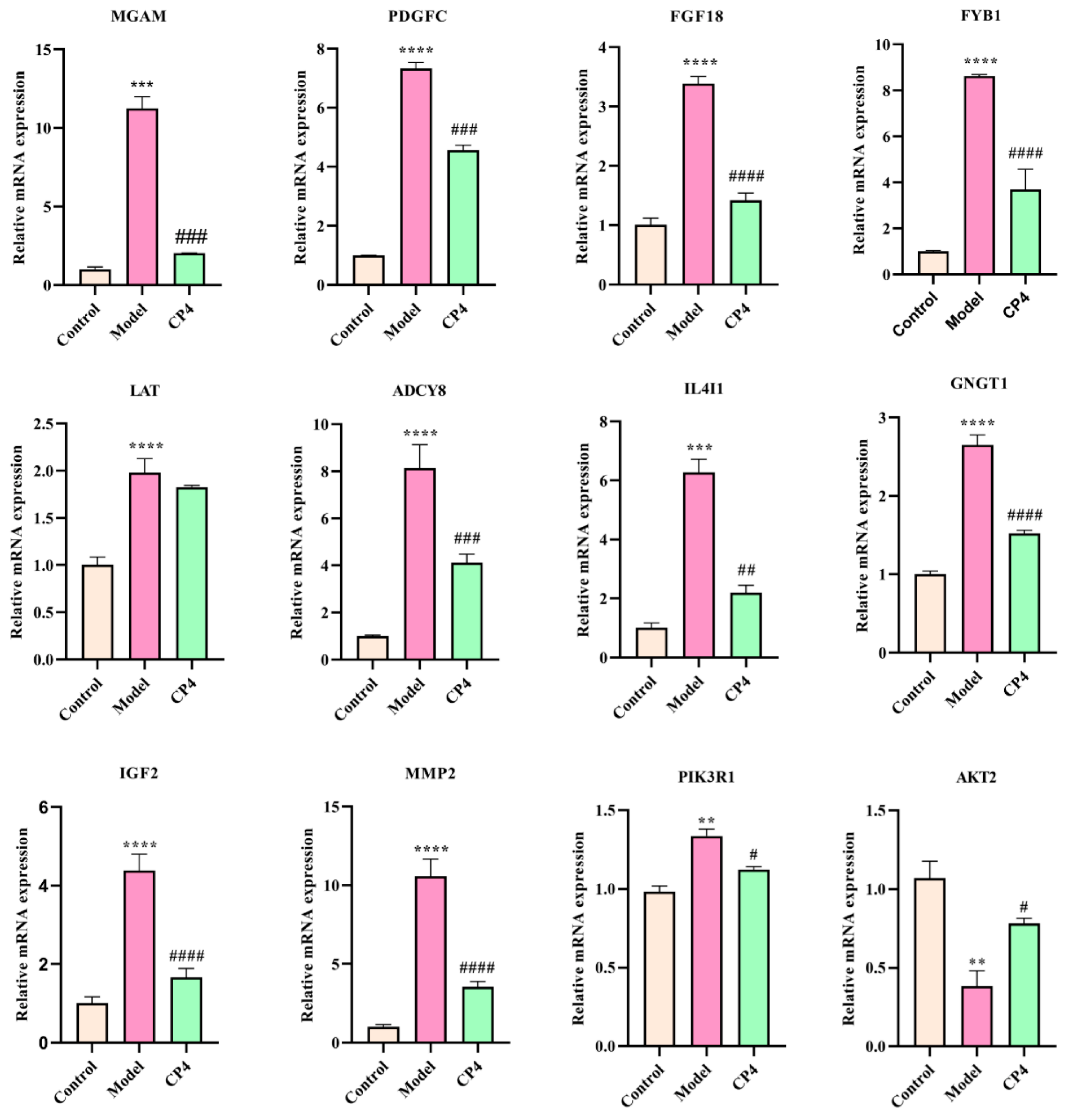

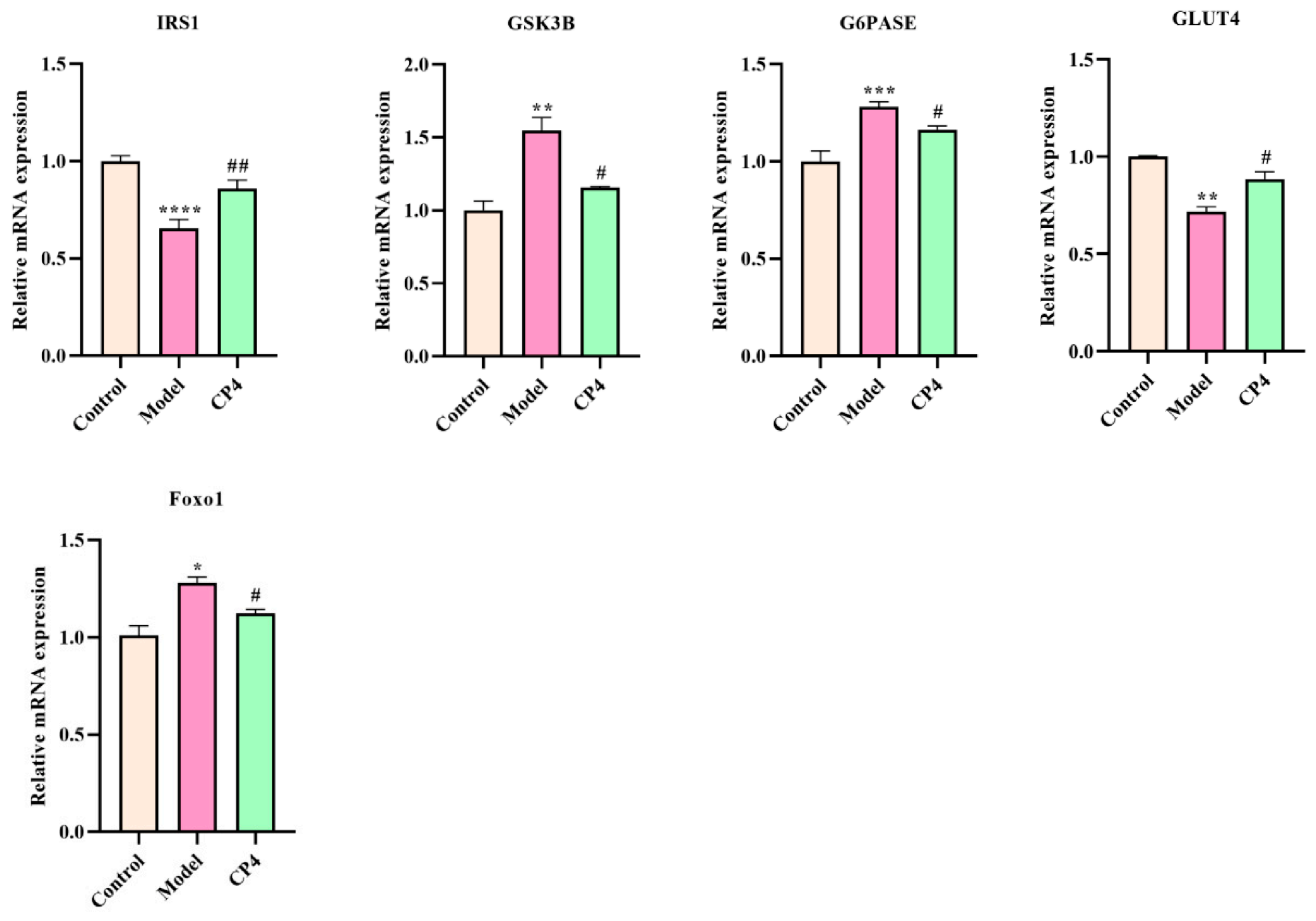
Effect of CP4 on the expression of key genes in IR-HepG2 cells. * *p* < 0.05, ** *p* < 0.01, *** *p* < 0.001, and **** *p* < 0.0001: compared with the control group. ^#^ *p* < 0.05, ^##^ *p* < 0.01, ^###^ *p* < 0.001, and ^####^ *p* < 0.0001: compared with the model group.

By integrating network pharmacology with transcriptomics, we further validated the expression profiles of multiple genes. CP4 treatment significantly suppressed the expression of MGAM, PDGFC, FGF18, FYB1, LAT, ADCY8, IL4I1, GNGT1, IGF2, MMP2, FOXO1, G6Pase, GSK3B, and IRS1, whilst upregulating GLUT4 and PIK3R1. These genes are associated with the PI3K-Akt, Rap1, AGE-RAGE (which is involved in diabetic complications), and Ras signaling pathways, all of which are linked to type 2 diabetes (Figure 12). Since insulin signaling is tightly regulated by protein phosphorylation and post-translational modifications, these changes in mRNA should be regarded as supportive molecular evidence and require further validation at the protein level.

## 4. Discussion

This study demonstrates that the donkey blood-derived peptide CP4 possesses both functional food-relevant properties and preliminary development potential as a nutraceutical candidate targeting insulin resistance (IR). From the perspective of functional ingredient development, the combination of low hemolytic activity, gastrointestinal digestive stability, inhibitory activities against α-glucosidase and pancreatic lipase, and antioxidant capacity collectively provides compelling evidence supporting its further evaluation as a bioactive compound for metabolic health applications. However, these data should be interpreted as early-stage evidence rather than direct proof of efficacy in humans.

The main novelty of the present work lies in the evaluation of a short donkey blood-derived peptide, YPWTQ, from an underutilized animal by-product and in the integration of cellular phenotypes with transcriptomic and metabolomic profiles. Therefore, CP4 should be positioned not as a completely new mechanistic class, but as a distinct peptide candidate with a specific source, sequence, and multi-functional in vitro profile.

At the cellular level, CP4 increased glucose consumption and glycogen content and reduced TG accumulation in IR-HepG2 cells. These effects are biologically relevant because impaired glucose utilization, reduced glycogen storage, and abnormal lipid accumulation are key features associated with hepatic insulin resistance. The RT-qPCR results suggested changes in genes linked to insulin signaling and glucose metabolism: following CP4 treatment, the expression of GLUT4, AKT2, and PIK3R1 was upregulated, while that of FOXO1, G6Pase, GSK3β, and IRS1 was downregulated. Consistent with previous studies, upregulation of GLUT4 enhances glucose uptake [33], while downregulation of GSK3β promotes glycogen synthesis and improves insulin sensitivity [34,35]. Previous research has shown that knocking out FOXO1 increases insulin sensitivity and partially alleviates insulin resistance (IR) [36]. In IR-HepG2 cells, Spexin downregulates the expression of phosphoenolpyruvate carboxykinase (PEPCK) and glucose-6-phosphatase (G6Pase), thereby alleviating IR-induced excessive hepatic glucose production [37]. Xia et al. [38] found that SMW and SMW-BI significantly increased the expression of GLUT2 and AKT2 in the livers of T2DM mice, further confirming that the hypoglycemic mechanism of SMW and SMW-BI is associated with AKT2. Concurrently, the upregulation of GCK expression in the transcriptome suggests that CP4 may promote the restoration of glucose metabolic homeostasis by increasing the expression of GCK, a rate-limiting enzyme in hepatic glucose metabolism, thereby helping to improve IR-related glucose metabolic disorders [39]. Collectively, these findings indicate that the anti-IR effects of CP4 are closely associated with the restoration of the PI3K-Akt-mediated glucose metabolic regulatory network.

At the same time, CP4 also shows potential for intervention in the regulation of lipid metabolism. At the cellular level, CP4 significantly reduced triglyceride (TG) accumulation in IR-HepG2 cells. Transcriptomic results indicate that CETP expression was downregulated following CP4 intervention. Previous studies have shown that CETP participates in lipid exchange processes between lipoproteins and is closely associated with the onset and progression of dyslipidemia [40,41]. Therefore, the downregulation of CETP suggests that CP4 may have a certain ameliorative effect on abnormal lipid transport and lipid metabolism imbalance. Compared to transcriptomics, metabolomics provides a more direct reflection of CP4’s regulatory focus at the level of lipid metabolism. Results show that following CP4 intervention, lipids and lipid-like molecules became the most significant differentially expressed metabolic categories, suggesting that the remodeling of lipid metabolism is a key mechanism by which CP4 alleviates IR. From the perspective of specific metabolic pathways, CP4 exerted significant effects on a variety of lipid-related metabolites. In the arachidonic acid metabolic pathway, CP4 significantly increased prostaglandin J2 levels. Prostaglandin J2 is a metabolite with anti-inflammatory properties that can reduce the inhibitory effect of chronic low-grade inflammation on insulin signaling by suppressing the excessive activation of the NF-κB inflammatory signaling pathway and thereby reducing the release of pro-inflammatory cytokines [42]. In the linoleic acid and unsaturated fatty acid metabolic pathways, CP4 promotes the upregulation of γ-linolenic acid, linoleic acid, and oleic acid, while downregulating eicosadienoic acid and 12,13-dihydroxyoleic acid (DiHOME). Previous studies have shown that γ-linolenic acid helps regulate fatty acid oxidation and lipid synthesis, thereby improving lipid metabolism disorders [43]; oleic acid has multi-target inhibitory effects and is associated with antidiabetic effects [44]; and oleic acid can enhance GLUT4 transport capacity in the PI3K pathway, alleviating metabolic damage under IR conditions to a certain extent [45]. Conversely, DiHOME, as a metabolite of linoleic acid produced by neutrophils and macrophages, may exhibit cytotoxicity if it accumulates excessively within cells, further exacerbating inflammatory responses and tissue damage [46]. Therefore, CP4’s regulation of these key metabolites indicates that it promotes the restoration of beneficial lipids while limiting the accumulation of harmful lipids, thereby reshaping the lipid metabolic environment to better support the maintenance of insulin sensitivity. Overall, CP4’s regulation of lipid metabolism is characterized by multiple targets and multiple levels. Its mechanism of action likely lies in correcting lipid metabolic imbalances, thereby providing a more favorable metabolic foundation for the restoration of insulin signaling and the reestablishment of glucose-lipid homeostasis.

Another important aspect of CP4’s action lies in its ability to alleviate oxidative stress in a state of insulin resistance. The decrease in malondialdehyde (MDA) and reactive oxygen species (ROS) levels in cells following CP4 treatment indicates that redox balance has been restored, which is crucial because oxidative stress is both a cause and a consequence of insulin resistance. Transcriptomic results further support these findings. The study found that following CP4 intervention, the expression of pro-inflammatory genes such as IL-4, IL-1, and TNF-α was downregulated, while CCR7 expression was upregulated. These transcriptional changes suggest a potential anti-inflammatory effect of CP4. At the same time, CP4 can also suppress the production of reactive oxygen species (ROS) and enhance antioxidant defense capabilities by downregulating HPX expression, thereby alleviating oxidative stress [47]. Therefore, the effects observed in this study may reflect the combined effects of antioxidant action and metabolic regulation, rather than a single, direct effect on insulin signaling. Future studies will need to further distinguish between these possibilities using methods such as pathway inhibitors, antioxidant control groups, and protein phosphorylation assays.

The intracellular localization of FITC-CP4 provides a spatial basis for explaining the cellular effects observed in this study. CP4 treatment increased the survival rate of IR-HepG2 cells, enhanced glucose consumption and glycogen content, and reduced triglyceride (TG) accumulation, reactive oxygen species (ROS) production, and malondialdehyde (MDA) levels. These results provide a spatial basis for how CP4 regulates intracellular glycolipid metabolism and oxidative stress status. This suggests that CP4, upon entering the cell, may be involved in the regulation of intracellular metabolism and the alleviation of oxidative stress. Meanwhile, RT-qPCR results showed that GLUT4, AKT2, and PIK3R1 expression were upregulated, while FOXO1, G6Pase, and GSK3β expression were downregulated, which is consistent with the phenotypic findings of enhanced glucose utilization and improved glycogen synthesis. However, fluorescence localization experiments only confirmed the cellular uptake and cytoplasmic distribution of CP4; they did not directly demonstrate the uptake efficiency, endocytic mechanism, or organelle-specific localization. Further studies using flow cytometry, time-dependent uptake experiments, endocytic inhibitors, and organelle colocalization markers are needed to elucidate the uptake kinetics and subcellular fate of CP4.

Several limitations should be emphasized. First, the study relied on a single glucosamine-induced IR-HepG2 model. HepG2 cells are convenient and retain certain hepatocyte-like functions, but they are tumor-derived and may differ from primary hepatocytes in insulin signaling, gluconeogenic response, and protein-expression pro-file. Second, no metformin or other insulin-sensitizing positive control was included in the cell experiments, which limits comparison with established antidiabetic interventions. Third, Oral bioavailability is a recognized barrier for many bioactive peptides [48]. Although simulated digestion suggested CP4 stability, no Caco-2 permeability, PepT1 transport, plasma stability, pharmacokinetic, or in vivo efficacy data are available. Fourth, the apparent stronger response at 64 μM than at 128 μM in some assays may reflect a non-linear peptide response, receptor/transporter saturation, peptide aggregation, or assay-specific variability, and should be further investigated using a broader concentration range.

Our laboratory is currently evaluating the efficacy of CP4 in alleviating type 2 diabetes (T2DM) by establishing a T2DM model. Preliminary blood glucose monitoring results indicate that CP4 has a good hypoglycemic effect. Overall, at this stage, CP4 should be regarded as a potential candidate worthy of further investigation, rather than a validated functional food ingredient. Future studies should include: validation of the IRS1/PI3K/AKT/GSK3β signaling pathway at the protein level; comparisons with metformin or other positive controls; determination of intestinal absorption and bioavailability; evaluation of CP4’s oral efficacy and safety; and systematic validation of these findings through the integration of data on blood glucose, insulin sensitivity, blood lipids, liver histology, and insulin signaling protein assays. Such studies are crucial for determining whether the in vitro effects observed in this paper can be translated into physiologically significant benefits.

From a translational research perspective, the development of CP4 requires the establishment of scalable and standardized production processes, an assessment of its allergenicity and safety, sensory evaluation, and an assessment of its stability during food processing and storage. Since CP4 is derived from donkey blood, consumer acceptance as well as cultural or dietary restrictions should also be taken into account. These issues are critical for any future CP4-based functional foods, dietary supplements, or nutritional health products.

## 5. Conclusions

In conclusion, the donkey blood-derived peptide CP4 showed low hemolytic activity, stability under simulated gastrointestinal digestion, α-glucosidase and pancreatic lipase inhibitory activity, and antioxidant capacity. In glucosamine-induced IR-HepG2 cells, CP4 partially improved glucose consumption and glycogen content while reducing TG accumulation, MDA, and ROS levels. Integrated transcriptomic, metabolomic, and RT-qPCR analyses suggested that these effects are associated with changes in genes and metabolites related to PI3K-Akt, Rap1, Ras, AGE-RAGE, and metabolic pathways. Collectively, these findings support the potential of CP4 as a food-derived bioactive peptide candidate for further investigation. However, protein-level validation, intestinal absorption and bioavailability studies, positive-control comparison, and in vivo experiments are still required before its efficacy, safety, and practical application as a functional food or nutraceutical ingredient can be confirmed.

## Figures and Tables

**Table 1:** High-Performance Liquid Chromatography (HPLC) detection parameter settings.

Chromatographic Parameters	Detailed Parameter Settings
Detection wavelength	280 nm
Column temperature	25 °C
Mobile phase flow rate	0.7 mL/min
Mobile phase A	A 2% aqueous acetonitrile solution containing 0.05% trifluoroacetic acid (TFA) (acetonitrile:water = 2:98, *v*/*v*)
Mobile phase B	A 90% aqueous acetonitrile solution containing 0.05% trifluoroacetic acid (TFA) (acetonitrile:water = 90:10, *v*/*v*)
Elution Protocol	0–15 min: 12% → 27% Phase B, linear gradient elution; 15–20 min: 27% Phase B, isocratic elution; 20–30 min: 27% → 12% Phase B, linear gradient elution.

**Table 2:** Primer sequences.

Gene Name	Primers
β-actin	F CATGTACGTTGCTATCCAGGC
	R CTCCTTAATGTCACGCACGAT
MGAM	F ACAGCCCGGTTGAAAAATCTG R CAGCAGCATTTCCACTGAAGG
PDGFC	F ATTCACAGCCCAAGGTTTCCT R GGGTCTTCAAGCCCAAATCTT
FGF18	F CACCAGCAAGGAGTGTGTGTT R CACCGTCGTGTACTTGAAGGG
FYB1	F GGATGTCTCAGTCAATAGCCG R GGTTCCTTGTCAGGCTTTTCC
LAT	F GATGAGGACGACTATCACAACCC R GAAGGCACTGTCTCGGATGC
ADCY8	F CAGGCAGTGCTATTCATGTGT R ACCTCCGAGTCTCCAGGAAAG
IL4I1	F GCCAAGACCCCTTCGAGAAAT R CCGATCCTGTTATCTGCCTCC
GNGT1	F ATTACGTTGAAGAACGATCTGGC R GGATGCCCTTTACCAGTGGA
IGF2	F GTGGCATCGTTGAGGAGTG R CACGTCCCTCTCGGACTTG
MMP2	F TACAGGATCATTGGCTACACACC R GGTCACATCGCTCCAGACT
IRS1	F AGCTCTGGTCGCCTTCTCTA R AGCTGTGTCCACCTTTCGAG
GLUT4	F ATAGGCTCCGAAGATGGGGA R CCCAGCCACGTCTCATTGTA
PIK3R1	F CAGAACACAGAGCTCCAGCA R TGAAAGCGTCAGCCAAAACG
AKT2	F GCCACCATGAATGAGGTGAA R GTACCCAATGAAGGAGCCGT
FOXO1	F GAGGGTTAGTGAGCAGGTTACA R TTGCTGCCAAGTCTGACGAA
G6Pase	F GGCTCTCAACTCCAGCATGT RAGGACGAGGGAGGCTACAAT
GSK3β	F CCTGGGAACTCCAACAAGGG R GGGGTCGGAAGACCTTAGTC

## Data Availability

The original contributions presented in this study are included in the article and Supplementary Materials. Further inquiries can be directed to the corresponding author.

## Supplementary Materials

The following supporting information can be downloaded at https://www.mdpi.com/article/10.3390/nu18152445/s1, Table S1: Details of Differentially Expressed Genes. Table S2: Details of Differentially Expressed Metabolites.

## Abbreviations

CP4	YPWTQ
DPP-IV	dipeptidyl peptidase-IV
T2DM	type 2 diabetes mellitus
IR	Insulin Resistance
ROS	Reactive oxygen species
MDA	Malondialdehyde
TG	Triglyceride
DAPI	4′,6-diamidino-2-phenylindole
BCA	bicinchoninic acid
GO	Gene Ontology
KEGG	Kyoto Encyclopedia of Genes and Genomes
GLP-1	Glucagon-like peptide-1
GlcN	Glucosamine
DN	diabetic nephropathy
PGJ2	prostaglandin J2
GLA	γ-linolenic acid
LA	linoleic acid
DiHOME	12,13-dihydroxyoleic acid
